# Repurposing Nirmatrelvir for Hepatocellular Carcinoma: Network Pharmacology and Molecular Dynamics Simulations Identify HDAC3 as a Key Molecular Target

**DOI:** 10.3390/ph18081144

**Published:** 2025-07-31

**Authors:** Muhammad Suleman, Hira Arbab, Hadi M. Yassine, Abrar Mohammad Sayaf, Usama Ilahi, Mohammed Alissa, Abdullah Alghamdi, Suad A. Alghamdi, Sergio Crovella, Abdullah A. Shaito

**Affiliations:** 1 Laboratory of Animal Research Center (LARC), Qatar University, Doha P.O. Box 2713, Qatar; m.suleman@qu.edu.qa; 2 Center for Biotechnology and Microbiology, University of Swat, Swat 19200, Pakistan; 3 Biomedical Research Center, QU Health, Qatar University, Doha P.O. Box 2713, Qatar; hyassine@qu.edu.qa; 4 Department of Biomedical Sciences at College of Health Sciences, QU Health, Qatar University, Doha P.O. Box 2713, Qatar; 5 School of Chemical Sciences, Universiti Sains Malaysia, Gelugor 10050, Penang, Malaysia; 6 Department of Medical Laboratory, College of Applied Medical Sciences, Prince Sattam bin Abdulaziz University, Al-Kharj 11942, Saudi Arabia; m.alissa@psau.edu.sa (M.A.); ab.alghamdi@psau.edu.sa (A.A.); s.alghamdi@psau.edu.sa (S.A.A.); 7 College of Medicine, QU Health, Qatar University, Doha P.O. Box 2713, Qatar

**Keywords:** HCC, HDAC3, network pharmacology, molecular docking, MD simulation

## Abstract

**Background:** Hepatocellular carcinoma (HCC) is one of the most common and fatal malignancies worldwide, characterized by remarkable molecular heterogeneity and poor clinical outcomes. Despite advancements in diagnosis and treatment, the prognosis for HCC remains dismal, largely due to late-stage diagnosis and limited therapeutic efficacy. Therefore, there is a critical need to identify novel therapeutic targets and explore alternative strategies, such as drug repurposing, to improve patient outcomes. **Methods:** In this study, we employed network pharmacology, molecular docking, and molecular dynamics (MD) simulations to explore the potential therapeutic targets of Nirmatrelvir in HCC. **Results:** Nirmatrelvir targets were predicted through SwissTarget (101 targets), SuperPred (1111 targets), and Way2Drug (38 targets). Concurrently, HCC-associated genes (5726) were retrieved from DisGeNet. Cross-referencing the two datasets identified 29 overlapping proteins. A protein–protein interaction (PPI) network constructed from the overlapping proteins was analyzed using CytoHubba, identifying 10 hub genes, with HDAC1, HDAC3, and STAT3 achieving the highest degree scores. Molecular docking revealed a strong binding affinity of Nirmatrelvir to HDAC1 (docking score = −7.319 kcal/mol), HDAC3 (−6.026 kcal/mol), and STAT3 (−6.304 kcal/mol). Moreover, Nirmatrelvir displayed stable dynamic behavior in repeated 200 ns simulation analyses. Binding free energy calculations using MM/GBSA showed values of −23.692 kcal/mol for the HDAC1–Nirmatrelvir complex, −33.360 kcal/mol for HDAC3, and −21.167 kcal/mol for STAT3. MM/PBSA analysis yielded −17.987 kcal/mol for HDAC1, −27.767 kcal/mol for HDAC3, and −16.986 kcal/mol for STAT3. **Conclusions:** The findings demonstrate Nirmatrelvir’s strong binding affinity towards HDAC3, underscoring its potential for future drug development. Collectively, the data provide computational evidence for repurposing Nirmatrelvir as a multi-target inhibitor in HCC therapy, warranting in vitro and in vivo studies to confirm its clinical efficacy and safety and elucidate its mechanisms of action in HCC.

## 1. Introduction

Liver cancer remains a major global health burden, with hepatocellular carcinoma (HCC) accounting for approximately 80–90% of all liver cancer cases. The incidence of liver cancer ranks sixth globally, while it stands third in cancer-related mortality, reflecting its high lethality [1,2,3,4]. Hepatocellular carcinoma (HCC) has become one of the leading causes of cancer-related deaths. The HCC age-standardized incidence rate (ASR) was 7.3 in 2018, with notable regional variations in incidence rates; Eastern Asia (ASR 14.8) and Northern Africa (ASR 13.2) had the highest incidence rates. HCC annual mortality rates are projected to more than double, increasing from 800,000 deaths in 2020 to an estimated 1.3 million per year by 2040 [4]. In the Middle East and North Africa (MENA) region, age-standardized mortality rates average 4.90 cases per 100,000 individuals annually, which is higher than the global average (3.42/100,000), primarily driven by rising etiological factors and a lack of access to appropriate preventive measures and therapies [5]. Cirrhosis remains the predominant risk factor for HCC, accounting for approximately 80% of the cases worldwide [5,6,7]. Other major risk factors contributing to hepatocellular carcinoma (HCC) include hepatitis B virus (HBV) and hepatitis C virus (HCV) chronic infections, exposure to aflatoxins, and the rising burden of Metabolic Dysfunction-Associated Steatohepatitis (MASH) due to increasing rates of obesity and type 2 diabetes. The existence of HBV and HCV antiviral therapies and vaccines has reduced HCC rates [8]. However, the growing global prevalence of MASH poses a new and urgent challenge, increasing HCC rates. In particular, MASH-related HCC can occur even in the absence of cirrhosis [9].

One of the most prominent barriers in HCC management is its early asymptomatic stage, often leading to a delayed diagnosis, when the disease is already advanced and virtually untreatable [1,10]. At the molecular level, HCC is highly heterogeneous, with overlapping subtype-specific molecular characteristics complicating treatment strategies. HCC’s diverse etiological factors further contribute to HCC heterogeneity. For example, patients with HBV-related HCC have different molecular features and immune response profiles compared to those with MASH-induced HCC [11,12].

Historically, curative approaches for early-stage HCC have centered on liver resection and transplantation. Non-surgical interventions such as local radiofrequency ablation and transarterial chemoembolization (TACE) have also been employed, particularly for early and intermediate stages [13]. The advent of targeted therapies and immunotherapies has, however, revolutionized the landscape of HCC treatment [14]. Sorafenib, a multikinase inhibitor targeting RAF, MEK, VEGFR, and PDGFR, was the first agent approved for advanced HCC following successful phase III trials. More recently, the IMbrave150 trial demonstrated that the combination of the PD-L1 inhibitor atezolizumab and the VEGF inhibitor bevacizumab significantly improved overall survival and progression-free survival compared to sorafenib [15,16]. In addition, new first- and second-line HCC drugs such as lenvatinib, durvalumab + tremelimumab have been introduced. Nevertheless, the best overall survival for these drugs is only 13.6 to 19.2 months, and their best objective response rates are 20 to 30% [17,18,19], substantiating the search for more effective treatment options, including drug discovery by repurposing existing drugs. In this context, network pharmacology (NP) introduces an innovative approach to drug discovery by transcending the limitations of traditional single-target methods. Through systems-level analysis of molecular interactions, NP helps uncover novel drug candidates, identify promising therapeutic targets, and reveal new uses for existing drugs. NP’s systems-level integrative framework holds particular promise for addressing the molecular complexity of diseases such as HCC [20].

At the molecular level, few actionable mutations exist in HCC [11,12]. Histone deacetylases (HDACs), particularly HDAC1 and HDAC3, modulate chromatin structure and gene expression, and their dysregulation contributes to oncogenesis [21]. Specifically, the dysregulation of HDAC1 leads to the repression of tumor suppressor genes and promotion of oncogenic pathways, contributing to tumorigenesis, while HDAC3, a class I HDAC, is also implicated in HCC progression through the modulation of apoptosis and immune responses [22]. In parallel, the Signal Transducer and Activator of Transcription 3 (STAT3) is constitutively activated in approximately 60% of HCC cases, promoting cell proliferation, angiogenesis, and immune evasion through the transcriptional activation of genes like Cyclin D and VEGF [23,24]. Persistent STAT3 activation often results from mutations of upstream genes, such as *JAK1* alterations or the loss of function of negative regulators like *SOCS1* and *SOCS3* [25]. The inhibition of STAT3 has been shown to induce apoptosis and suppress tumor growth in preclinical models [26]. Given these challenges, drug repurposing emerges as a promising strategy to accelerate therapeutic development in cancer and other diseases [27].

Nirmatrelvir, a key component of the FDA-approved antiviral Paxlovid, was initially developed to target the SARS-CoV-2 main protease (Mpro, also called 3CL) [28,29]. Recent studies suggest that it may exhibit immunomodulatory and antitumor potential, including activity on IL-6 and related pathways [30,31]. However, its role in HCC has not been previously explored.

Therefore, to provide a mechanistic and robust computational rationale for repurposing Nirmatrelvir in HCC, we employed an integrative computational approach that combines network pharmacology, molecular docking, and molecular dynamics simulations to systematically investigate the potential molecular targets of Nirmatrelvir in HCC.

## 2. Results and Discussion

### 2.1. Nirmatrelvir Targets and HCC-Associated Target Identification

**Figure 1:**
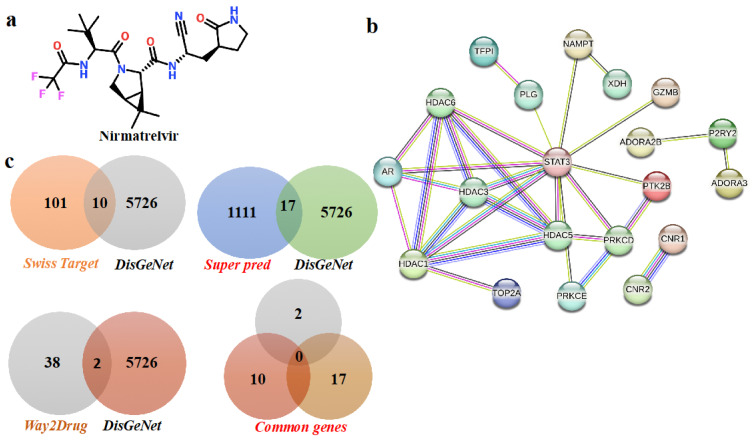
The identification of overlapping targets between Nirmatrelvir and hepatocellular carcinoma (HCC). A Venn diagram was generated to illustrate the intersection between the predicted molecular targets of Nirmatrelvir and the disease-associated genes linked to HCC. Nirmatrelvir targets were retrieved from SwissTargetPrediction and SEA databases, while HCC-related genes were compiled from GeneCards, DisGeNET, and OMIM. The resulting overlap represents the common genes that may mediate the therapeutic effects of Nirmatrelvir in the context of liver cancer. Disease and Nirmatrelvir-associated common targets: (**a**) the two-dimensional structure of Nirmatrelvir. (**b**) The protein–protein interaction network of common targets. (**c**) The common targets according to Venn diagrams.

**Figure 2:**
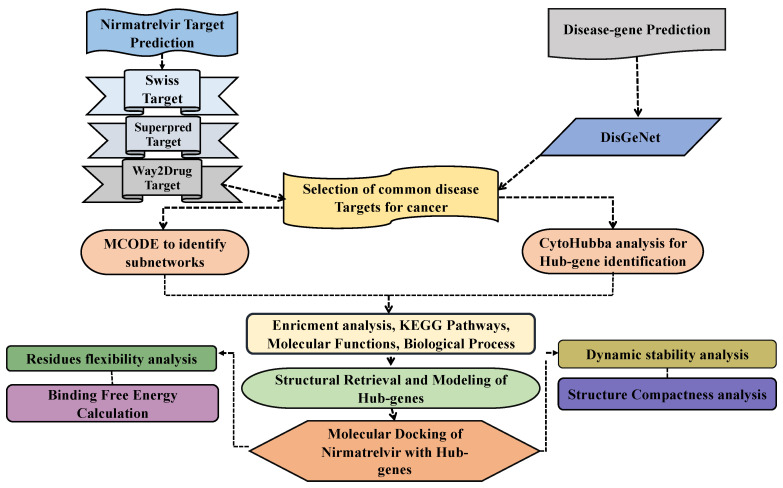
A schematic overview of the study workflow integrating network pharmacology and computational drug discovery approaches. The analysis begins with the identification of potential molecular targets of the drug Nirmatrelvir using multiple target prediction platforms. In parallel, disease-associated genes related to hepatocellular carcinoma (HCC) are retrieved from curated public databases. The two datasets are then compared to identify overlapping targets, representing the possible mechanistic intersection between Nirmatrelvir and HCC. These common targets are subsequently used to construct a protein–protein interaction (PPI) network to uncover functional interconnectivity. Hub genes within the network are identified based on topological parameters to pinpoint the most central and biologically relevant nodes. Selected hub genes are subjected to molecular docking with Nirmatrelvir to evaluate binding affinity and interaction stability. Finally, molecular dynamics simulations are conducted for the top-performing gene–drug complexes to assess their conformational stability, dynamic behavior, and binding persistence in a physiological environment. This in silico pipeline aims to reveal novel therapeutic insights into the repurposing potential of Nirmatrelvir for liver cancer treatment.

To explore how Nirmatrelvir (Figure 1a) engages with key targets in hepatocellular carcinoma, a combination of computational tools and databases was utilized, as illustrated in the workflow in Figure 2. Nirmatrelvir target predictions yielded 101 targets from SwissTargetPrediction, 1111 targets from SuperPred, and 38 targets from Way2Drug. Concurrently, disease-associated genes for liver cancer were identified using DisGeNet, revealing 5726 genes linked to HCC. A comparison of these datasets revealed overlapping targets: 10 genes were common to both SwissTargetPrediction and DisGeNet, 17 genes were shared between SuperPred and DisGeNet, and 2 genes were found in both Way2Drug and DisGeNet datasets. Altogether, 29 genes were identified as common to Nirmatrelvir and liver cancer (Figure 1c). The 29 overlapping targets were then used to construct a protein–protein interaction (PPI) network (Figure 1b).

### 2.2. Identification of Hub Genes and Small Subnetworks 

**Figure 3:**
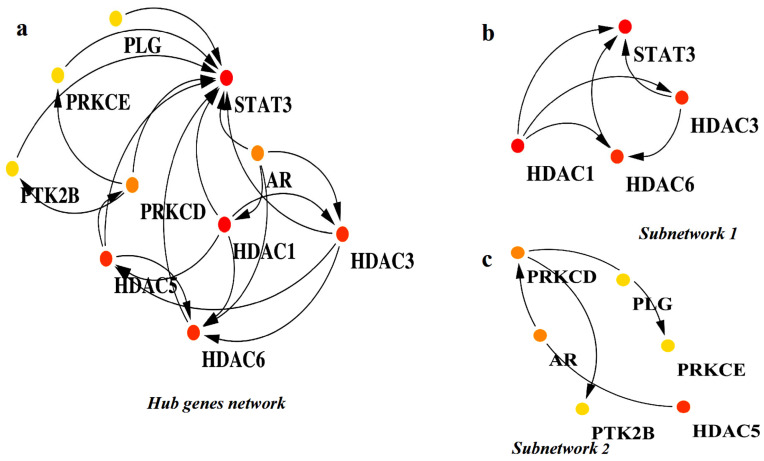
The identification of hub genes and key subnetworks within the PPI network of overlapping targets. (**a**) The visualization of the entire protein–protein interaction (PPI) network constructed from overlapping genes. Nodes represent proteins, with hub genes highlighted using a color gradient: red (the top 5 highest degree nodes), orange (the next 5), and yellow (the subsequent top 5). (**b**) Subnetwork 1 is a densely connected module extracted from the main PPI network, featuring hub genes and their immediate interactors. (**c**) Subnetwork 2 is another functional module showing a distinct cluster of hub gene interactions relevant to key biological processes.

Analyzing subnetworks within PPI networks using tools such as MCODE is crucial in systems biology, as it helps uncover the functional structure of biological systems and provides valuable insights into disease mechanisms by highlighting important molecular interactions [32,33]. In this study, we applied the MCODE algorithm to explore the subnetworks within the PPI network of the common target proteins. This analysis resulted in the identification of two distinct subnetworks. The subnetwork 1, illustrated in Figure 3b, includes the target proteins STAT3, HDAC1, HDAC3, and HDAC6. The subnetwork2, shown in Figure 3c, comprises HDAC5, PRKCE, PTK2B, AR, PLG, and PRKCD. Furthermore, to identify the hub genes within the PPI network, we utilized the CytoHubba plugin in Cytoscape. Based on the degree score, the analysis highlighted ten hub genes: STAT3, PRKCE, PLG, PTK2B, PRKCD, AR, HDAC5, HDAC1, HDAC3, and HDAC6 (Figure 3a). Among these genes, the top three, HDAC1, HDAC3, and STAT3, were selected as significant biomarkers due to their high degree scores. STAT3, while normally transiently activated, exhibits abnormal and sustained activation in several solid and hematologic malignancies, including melanoma, multiple myeloma, and cancers of the breast, prostate, ovary, and colon [34]. HDAC1, a key epigenetic regulator, inhibits the acetylation of both histone and non-histone proteins [35]. Although it is notably overexpressed in lung cancer, the relationship between HDAC1 expression and the clinical outcomes of lung cancer patients remains unclear [36,37]. HDAC3, on the other hand, plays roles in apoptosis, cell cycle regulation, and DNA repair. Its overexpression has been linked to a wide range of cancers, including leukemia, melanoma, lymphoma, epithelial cancers, hepatocellular carcinoma, pancreatic cancer, gastric cancer, and cancers of the liver, breast, prostate, and colon [38]. The consistent involvement of these hub genes in multiple cancer types underscores their potential as promising therapeutic targets, particularly in the context of HCC.

### 2.3. Analysis of the KEGG Pathway and Gene Ontology (GO) Function Enrichment

We conducted enrichment analysis on the top 10 hub genes using the DAVID database, focusing on key aspects such as molecular function, biological processes, cellular components, and KEGG pathway involvement. The analysis prioritized terms based on their statistical significance, with the false discovery rate (FDR) serving as the primary threshold for identifying meaningful associations. The GO biological processes analysis revealed the distribution of our proteins in protein deacetylation, the epigenetic regulation of gene expression, MAPK cascade, the positive regulation of signaling receptor activity, protein phosphorylation, the regulation of primary metabolic processes, the regulation of sequestered calcium ion release into cytosol, the positive regulation of superoxide anion generation, the regulation of multicellular organism growth, and responses to immobilization stress and intracellular receptor signaling pathways, as shown in Table 1. The GO cellular component analysis indicated that the identified proteins were localized across various subcellular structures, including the cytosol, cytoplasm, nucleus, nucleoplasm, plasma membrane, and Golgi apparatus. Likewise, the GO molecular function analysis showed that the proteins participate in a range of activities, such as enzyme binding, protein lysine deacetylase activity, histone deacetylase activity, transcription corepressor binding, tubulin deacetylase activity, histone deacetylase binding, and DNA-binding transcription factors. Finally, KEGG pathway analysis demonstrated that the identified proteins are associated with several critical cellular signaling routes, including viral carcinogenesis, microRNAs in cancer, the AGE-RAGE signaling pathway in diabetic complications, insulin resistance, the chemokine signaling pathway, type II diabetes mellitus, the GnRH signaling pathway, inflammatory mediators regulating TRP channels, Fc gamma R-mediated phagocytosis, and pathways in cancer. The analysis is shown in Table 1.

### 2.4. Survival and Expression Analysis of Hub Genes

**Figure 4:**
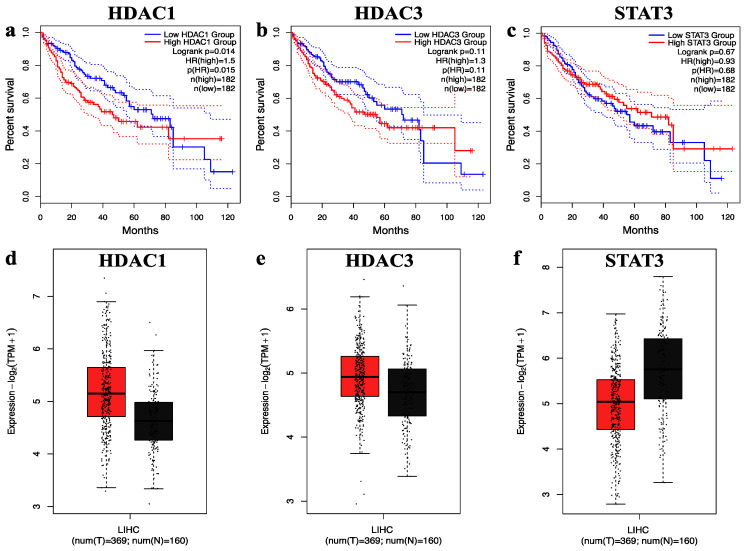
The overall survival and expression analysis of selected hub genes. (**a**–**c**) The overall survival of HDAC1, HDAC3, and STAT3 genes in hepatocellular carcinoma. (**d**–**f**) The relative expression levels of HDAC1, HDAC3, and STAT3 genes in hepatocellular carcinoma shown by the red and black boxes, which correspond to the tumor and normal sample, respectively.

GEPIA, an interactive web-based platform, enables comprehensive analysis of RNA sequencing data from the TCGA and GTEx databases. It is widely employed in cancer research for exploring gene expression patterns, including differential expression and survival analysis [39]. In our study, we utilized GEPIA’s survival analysis tool to assess the prognostic significance of selected hub genes. Figure 4 highlights the selected hub genes HDAC1, HDAC3, and STAT3, demonstrating their correlation with overall survival outcomes and expression differences between cancerous and normal tissues. Kaplan–Meier survival curves (Figure 4a–c) compare patients with high (red) versus low (blue) expression levels of each gene, with time in months on the x-axis and survival probability on the y-axis. Notably, elevated expression levels of these genes were associated with poorer overall survival. In addition, gene expression box plots (Figure 4d–f) illustrate the expression profiles of HDAC1, HDAC3, and STAT3 in normal liver tissues (n = 160) and hepatocellular carcinoma samples (n = 369). The analysis revealed that HDAC1 and HDAC3 were significantly overexpressed in cancerous tissues compared to normal samples, reinforcing their potential role in tumorigenesis. Overall, these results underscore the clinical relevance of HDAC1, HDAC3, and STAT3 as potential prognostic markers and therapeutic targets in hepatocellular carcinoma.

### 2.5. Molecular Docking Analysis of Nirmatrelvir and Selected Target Proteins

**Figure 5:**
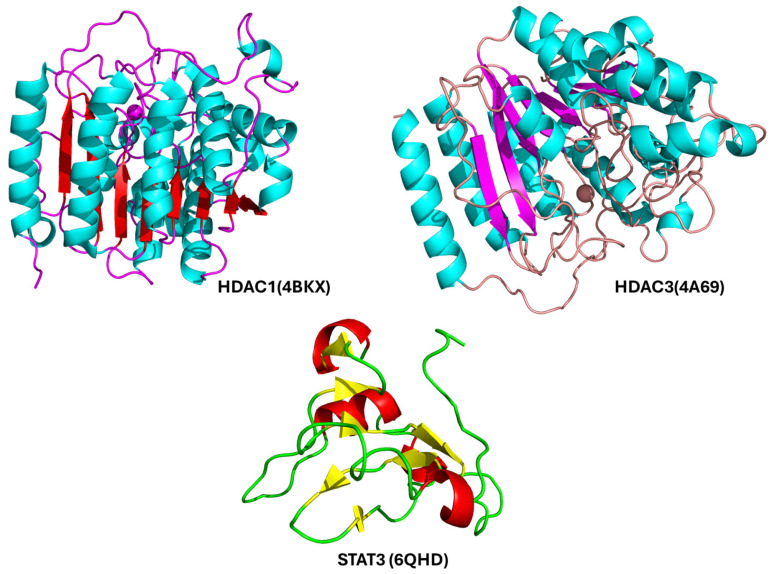
Ribbon models of the three key protein targets prioritized for molecular docking with Nirmatrelvir: HDAC1 (Histone Deacetylase 1), a zinc-dependent enzyme involved in chromatin remodeling and transcriptional repression, commonly overexpressed in HCC; HDAC3 (Histone Deacetylase 3), another class I HDAC with critical roles in epigenetic regulation, inflammation, and cancer progression; the SH2 (Src Homology 2) domain of STAT3 (Signal Transducer and Activator of Transcription 3), which mediates phosphorylation-dependent dimerization and the activation of STAT3, a transcription factor frequently dysregulated in liver cancer.

Molecular docking serves as a crucial in silico technique for investigating the interaction between drug candidates and target proteins. It provides a detailed understanding of how a compound may align within a protein’s active site, assessing both the strength and nature of the interaction. In this research, docking simulations were utilized to examine how effectively Nirmatrelvir can associate with the identified key proteins, offering insights into its therapeutic potential [40]. To perform the docking analysis, the crystal structures of the target proteins HDAC1 (PDB ID: 4BKX), HDAC3 (PDB ID: 4A69), and the SH2 domain of STAT3 (PDB ID: 6QHD) were retrieved from the Protein Data Bank. Binding site residues were selected based on evidence from previous studies to ensure accuracy in docking predictions. The 3D conformations of these target proteins used in the docking simulations are illustrated in Figure 5.

The docking results of Nirmatrelvir against HDAC1, HDAC3, and STAT3 provide insights into its potential inhibitory effects on these targets. The detailed interaction analysis from the docking studies is summarized in Table 2 and reveals multiple hydrogen bond (HB) interactions for the Nirmatrelvir functional groups involved in interactions, and their corresponding amino acid residues, interaction types (hydrogen bonding), and bond distances (Å).

**Figure 6:**
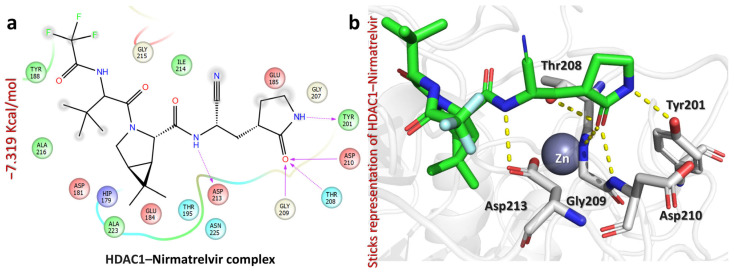
The figure shows how Nirmatrelvir interacts with the HDAC1 target. (**a**) The 2D interaction pattern of Nirmatrelvir with HDAC1. (**b**) The 3D interaction pattern of Nirmatrelvir with HDAC1. The most relevant amino acid positions are shown.

Among the three complexes, Nirmatrelvir exhibited the strongest binding affinity towards HDAC1, with a docking score of −7.319 kcal/mol. The interaction profile revealed multiple hydrogen bonds, particularly with Tyr201, Thr208, Gly209, Asp210, and Asp213, suggesting the strong stabilization of the ligand within the binding site (Figure 6). Specifically, the carbonyl of pyrrolidin-2-one interacts with Tyr201 (2.06 Å), the carbonyl of pyrrolidin-2-one forms hydrogen bonds with Thr208 (2.39 Å), Gly209 (2.10 Å), and Asp210 (2.17 Å), and the NH of amides interacts with Asp213 (2.00 Å). These interactions indicate the high likelihood of the effective inhibition of HDAC1, which plays a critical role in gene expression regulation through histone deacetylation. Strong binding to HDAC1 suggests that Nirmatrelvir could potentially modulate epigenetic pathways, making it a promising candidate for further exploration in cancer therapy.

**Figure 7:**
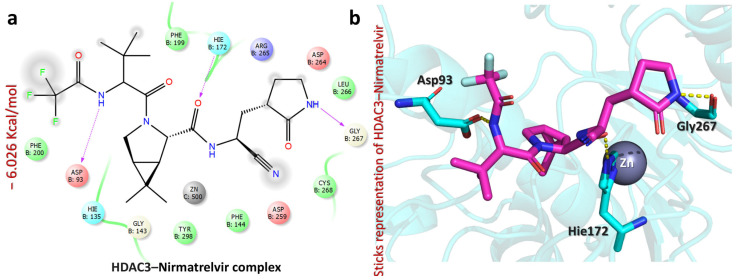
The figure shows the bonding network between Nirmatrelvir and the HDAC3 protein. (**a**) The 2D interaction pattern of Nirmatrelvir with HDAC3. (**b**) The 3D interaction pattern of Nirmatrelvir with HDAC3. The most relevant amino acid positions are shown.

Furthermore, the HDAC3–Nirmatrelvir complex revealed a docking score of −6.026 kcal/mol, suggesting a notable interaction between the ligand and the protein active site (Figure 7). A closer look at the binding interactions highlights key stabilizing forces within the complex with residues like Asp93, Hie172, and Gly267 (Figure 7) [41]. The carbonyl of trifluoroacetamide forms a strong hydrogen bond with Asp93 (1.92 Å), while the carbonyl of cyanoethylacetamide interacts with Hie172 (1.93 Å), and the NH of pyrrolidin-2-one establishes a hydrogen bond with Gly267 (2.05 Å) (Figure 7). These interactions indicate that Nirmatrelvir engages with HDAC3 through a network of hydrogen bonds, potentially influencing protein activity. Further computational studies, such as molecular dynamics simulations and free energy calculations, would be valuable to understand the stability and long-term interaction of Nirmatrelvir within the HDAC3 binding site.

**Figure 8:**
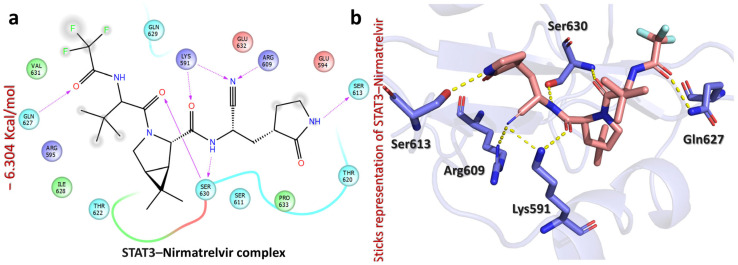
The figure shows the bonding network of the Nirmatrelvir–STAT3 complex. (**a**) The 2D interaction pattern of Nirmatrelvir with STAT3. (**b**) The 3D interaction pattern of Nirmatrelvir with STAT3. The most relevant amino acid positions are shown.

Moreover, Nirmatrelvir–STAT3 exhibited a docking score of −6.304 kcal/mol, indicating its moderate binding affinity. The ligand formed multiple hydrogen bonds with key residues, suggesting the potential inhibition of STAT3 activity (Figure 8). Specifically, the carbonyl of cyanoethylacetamide interacts with Lys591 (2.05 Å), while the nitrogen of cyanoethylacetamide forms hydrogen bonds with Lys591 (2.41 Å), Arg609 (2.24 Å), and Ser630 (1.83 Å). Additionally, the carbonyl of amide interacts with Ser630 (2.31 Å), the NH of pyrrolidin-2-one interacts with Ser613 (1.92 Å), and the carbonyl of trifluoroacetamide forms a hydrogen bond with Gln627 (1.74 Å). Notably, interactions with Lys591 and Arg609 suggest that Nirmatrelvir might interfere with STAT3’s dimerization or DNA-binding activity [42], which could be beneficial for targeting STAT3-driven liver cancers and inflammatory diseases [43]. Overall, Nirmatrelvir exhibited the strongest potential for HDAC1, HDAC3, and STAT3 inhibition, forming a well-defined interaction network with key catalytic residues, suggesting its role as a potent modulator of their activity. To thoroughly assess Nirmatrelvir’s inhibitory potential against these targets, additional molecular dynamics simulations and free energy calculations were conducted, providing a detailed understanding of its interactions.

Notably, our computational analyses revealed a notable affinity of HDAC1 and HDAC3, which are key epigenetic regulators in HCC, to Nirmatrelvir, which was originally developed as a specific peptidomimetic inhibitor of the SARS-CoV-2 Mpro protease. This apparent cross-activity may be explained by structural and mechanistic similarities between the active sites of viral proteases and class I HDACs. Both protein families feature a narrow catalytic tunnel and include coordination with a zinc ion as part of their mechanism of action [22,44]. HDAC1 and HDAC3 contain conserved catalytic domains with deep, zinc-coordinated active sites essential for deacetylase function [22,45]. Mpro features a similarly recessed substrate-binding cleft, though it utilizes a cysteine–histidine catalytic dyad (Cys145-His41) instead of a metal ion [44].

Despite differences in their catalytic chemistry, the geometric and steric similarity of these active sites permits partial overlap in ligand recognition. Nirmatrelvir’s electrophilic nitrile warhead and flexible scaffold enable high-affinity binding within proteolytic clefts and may likewise facilitate reversible interactions with the zinc-bound residues or adjacent histidine–aspartate motifs in HDACs. This structural convergence suggests a plausible mechanistic basis for the off-target epigenetic modulation of HDACs by Nirmatrelvir [22,44,45].

This structural convergence suggests that viral protease inhibitors, such as Nirmatrelvir, may exert off-target effects on zinc-dependent epigenetic regulators. Accordingly, the HDAC-inhibitory potential of Nirmatrelvir offers a rational, testable strategy for the repurposing of drugs in HCC. Such polypharmacological potential warrants further experimental validation and may lead to new epigenetic therapeutic avenues in liver cancer management.

### 2.6. Simulation of Dynamic Stability in Nirmatrelvir–Protein Complexes

**Figure 9:**
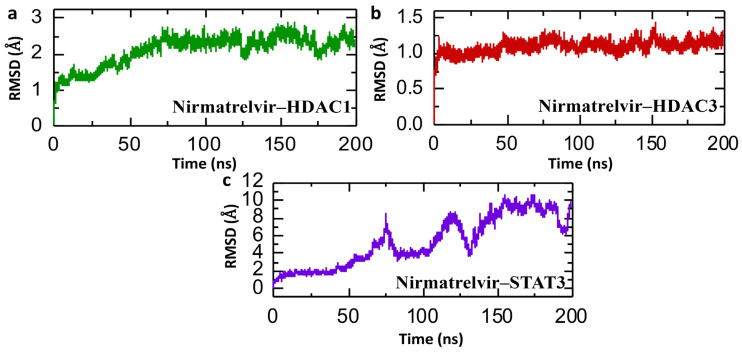
Dynamic stability simulation of Nirmatrelvir–protein complexes. (**a**) The RMSD trajectories for the Nirmatrelvir–HDAC1 complex. (**b**) The RMSD trajectories for the Nirmatrelvir–HDAC3 complex. (**c**) The RMSD trajectories for the Nirmatrelvir–STAT3 complex throughout a 200 ns simulation.

Studying consistent dynamic patterns is useful for determining how effectively ligands interact in simulations, which is critical for understanding pharmaceutical efficacy and target interaction stability. Calculating the Root Mean Square Deviation (RMSD) over time helps to assess the drug’s long-term stability in the active site of target proteins. This calculation assists in monitoring any changes or fluctuations in the complex’s structure during the simulation, enabling us to determine its stability [46]. The post-simulation RMSD (Root Mean Square Deviation) analysis presented for the Nirmatrelvir–protein complexes over a 200 ns molecular dynamics trajectory provides insights into the conformational stability and binding dynamics of Nirmatrelvir with HDAC1, HDAC3, and STAT3. In Figure 9a, the Nirmatrelvir–HDAC1 complex begins with a low RMSD near 0.5 Å and exhibits a gradual increase, stabilizing after ~75 ns in the range of 2.0–2.5 Å. This suggests an initial adaptation phase, possibly due to conformational rearrangement or binding pocket accommodation, followed by equilibrium with moderate structural fluctuations until the end of the simulation. Similarly, Figure 9b shows a Nirmatrelvir–HDAC3 complex with a consistently low RMSD (~1.0 Å) throughout the simulation, indicating a highly stable interaction with minimal structural deviation from the initial conformation. This suggests that Nirmatrelvir fits well within the HDAC3 binding pocket and maintains a rigid binding mode. Repeating the simulations for both HDAC1 and HDAC3 yielded comparable RMSD profiles, supporting the reproducibility of the observed binding stability. In contrast, Figure 9c reveals a highly fluctuating RMSD trajectory for the Nirmatrelvir–STAT3 complex for the case of repeat 1, with values increasing progressively to a peak of nearly 10 Å and exhibiting multiple sharp deviations throughout the simulation. However, in the case of repeat 2, the complex shows a highly stable RMSD value. This indicates significant structural rearrangements and instability, implying that the complex may not have a well-defined binding mode or that Nirmatrelvir binding induces substantial conformational changes in STAT3. Overall, the RMSD profiles suggest that Nirmatrelvir forms a stable and favorable complex with HDAC1 and HDAC3, and a highly unstable and possibly transient interaction with STAT3, highlighting potential differences in binding affinity and structural compatibility among the targets. Given the critical roles of HDAC1 and HDAC3 in epigenetic regulation and tumor progression in HCC, the stable binding of Nirmatrelvir to these targets indicates potential therapeutic relevance.

### 2.7. Post-Simulation Structural Compactness Analysis of Nirmatrelvir–Protein Complexes

**Figure 10:**
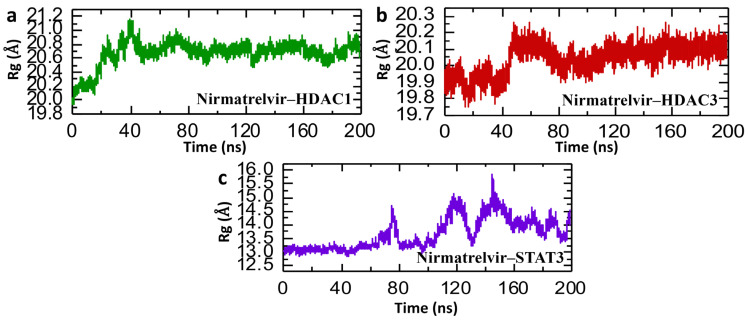
Post-simulation structural compactness analysis of Nirmatrelvir–protein complexes. (**a**) The compactness of the Nirmatrelvir–HADC1 complex as Rg. (**b**) The compactness of the Nirmatrelvir–HDAC3 complex as Rg. (**c**) The compactness of the Nirmatrelvir–STAT3 complex as Rg throughout the 200 ns simulation.

In molecular dynamics simulations, the Radius of Gyration (Rg) offers insight into the overall structural compactness of a protein–ligand complex. A stable or slightly decreasing Rg over the course of the simulation suggests that the complex maintains its structural integrity, reflecting a well-packed and consistent interaction interface. Such behavior not only points to the physical stability of the complex but may also imply that the binding interaction is strong and potentially therapeutically relevant [47]. Therefore, we analyzed the compactness of the Nirmatrelvir and selected hub genes and calculated the Rg value. Figure 10 presents the Radius of Gyration (Rg) analysis of Nirmatrelvir in complexes with HDAC1, HDAC3, and STAT3 over a 200 ns molecular dynamics simulation, offering insights into the structural compactness and folding behavior of the protein–ligand complexes. In Figure 10a, the Nirmatrelvir–HDAC1 complex shows an initial rise in Rg from ~20.0 Å to ~20.8 Å within the first 40 ns, followed by a relatively stable phase at around 20.6–20.8 Å, indicating a modest expansion of the protein structure early on and subsequent stabilization. However, Figure 10b for the Nirmatrelvir–HDAC3 complex reveals a slight but consistent increase in Rg from ~19.8 Å to ~20.2 Å within the first 50 ns, stabilizing thereafter with mild fluctuations, suggesting good compactness with minor conformational flexibility. The repetition of the simulation for both HDAC1 and HDAC3 showed similar Rg profiles, demonstrating the reproducibility of the results. In contrast, Figure 10c shows pronounced Rg fluctuations of the Nirmatrelvir–STAT3 complex for the case of repeat 1, ranging from ~13.0 Å to nearly 15.5 Å throughout the simulation, with multiple peaks and drops. This indicates dynamic structural rearrangements and poor compactness, reflective of instability or the partial unfolding of the protein upon ligand binding. However, in the case of Nirmatrelvir–STAT3 repeat 2, the pattern of Rg is completely altered, showing a highly compact pattern compared to repeat 1. In conclusion, the Rg data support and reinforce the RMSD findings. Nirmatrelvir–HDAC3 exhibits both a low RMSD and stable Rg values, indicating a highly stable and compact complex suitable for potential therapeutic targeting in hepatocellular carcinoma (HCC). Nirmatrelvir–HDAC1 shows moderate increases in both RMSD and Rg, reflecting a stable but more flexible interaction. Conversely, Nirmatrelvir–STAT3 shows large fluctuations in both RMSD and Rg, confirming a structurally unstable and dynamically disordered complex.

### 2.8. Post-Simulation Residual Fluctuation Analysis of Nirmatrelvir–Protein Complexes

**Figure 11:**
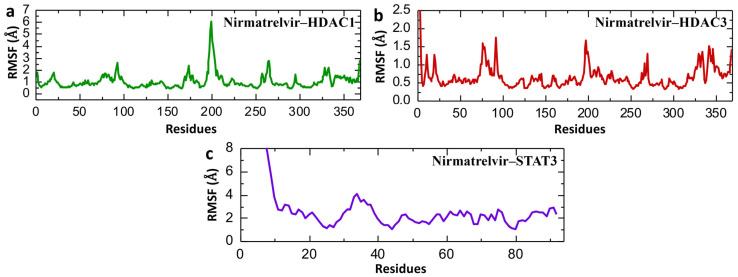
Post-simulation residual fluctuation analysis of Nirmatrelvir–protein complexes. (**a**) The residual fluctuation of the Nirmatrelvir–HADC1 complex. (**b**) The residual fluctuation of the Nirmatrelvir–HADC3 complex. (**c**) The residual fluctuation of the Nirmatrelvir–STAT3 complex during the 200 ns simulation.

Root Mean Square Fluctuation (RMSF) analysis serves as a key indicator of the flexibility and mobility of specific regions within a biomolecule or between interacting molecules during molecular dynamics (MD) simulations. It helps pinpoint flexible segments that may be critical for ligand binding or protein–protein interactions. Additionally, RMSF is instrumental in validating the reliability of MD simulations by comparing simulated fluctuation profiles with those obtained from experimental data [48,49]. Figure 11 presents the Root Mean Square Fluctuation (RMSF) analysis of individual amino acid residues for Nirmatrelvir in complex with HDAC1, HDAC3, and STAT3, providing insight into local flexibility and dynamic regions within the protein structures over the course of the 200 ns simulation. Nirmatrelvir–HDAC1 exhibits moderate fluctuations with peaks around residues at ~80, ~200, and ~260, and a significant spike near residue 200 reaching ~6.5 Å (Figure 11a), indicating localized flexibility in the loop region, while most of the structure remains below 2 Å, suggesting general structural rigidity with specific flexible loops. Similarly, Figure 11b shows an overall lower RMSF profile of Nirmatrelvir–HDAC3 and Nirmatrelvir–HDAC1, with values mostly below 1 Å and only minor spikes near residues 90, 200, 270, and 350, reflecting a more uniformly stable structure with fewer flexible regions. In contrast, Nirmatrelvir–STAT3 shows drastically different behavior, with RMSF values reaching above 7 Å at the N-terminus and maintaining high variability throughout the structure, particularly in the first 40 residues (Figure 11c), highlighting substantial local instability and disorder in the STAT3 complex upon Nirmatrelvir binding. The repetition of the simulation for both HDAC1, HDAC3, and STAT3 showed consistent RMSF profiles, confirming the reproducibility of the results. The RMSF data align well with both RMSD and Rg findings. HDAC3 consistently exhibits the lowest residue-level fluctuations, overall RMSD, and compact Rg values, confirming its highly stable interaction with Nirmatrelvir. HDAC1 displays moderate fluctuations and flexibility, especially in certain loop regions, which is consistent with its moderate RMSD and Rg profiles. In contrast, STAT3 shows high RMSF peaks, mirroring the large RMSDs and Rg instability identified, signifying a lack of structural integrity and stable binding with Nirmatrelvir.

### 2.9. Post-Simulation Analysis of Hydrogen Bonding in Nirmatrelvir–Protein Complexes

**Figure 12:**
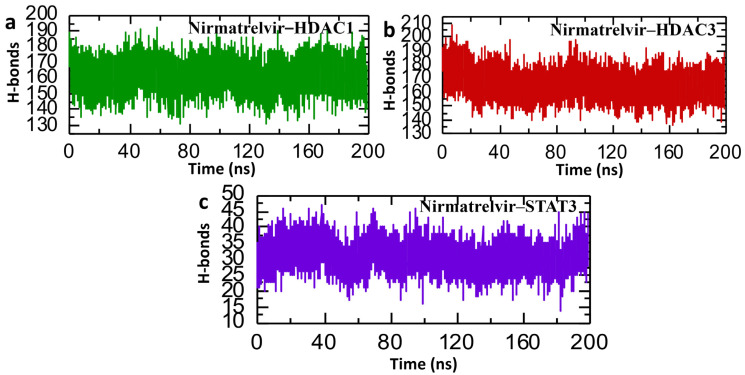
Post-simulation bonding network analysis of Nirmatrelvir–protein complexes. (**a**) The average hydrogen bonds in the Nirmatrelvir–HDAC1 complex. (**b**) The average hydrogen bonds in the Nirmatrelvir–HDAC3 complex. (**c**) The average hydrogen bonds in the Nirmatrelvir–STAT3 complex.

In the context of post-simulation analysis, hydrogen bonds play a critical role in determining the stability and strength of protein–ligand interactions. Monitoring these bonds throughout molecular dynamics simulations provides valuable insights into how binding affinity is regulated over time. This information helps elucidate key biological mechanisms, the molecular basis of disease progression, and the impact of genetic mutations on protein interaction networks and signaling pathways. Therefore, to check the binding stability of the Nirmatrelvir drug with selected target proteins, we calculated the average of post-simulation hydrogen bonds. The post-simulation hydrogen bond analysis of the Nirmatrelvir–protein complexes (HDAC1, HDAC3, and STAT3) over a 200 ns molecular dynamics simulation revealed insights into the stability and interaction dynamics of the drug with each target. For the Nirmatrelvir–HDAC1 complex, the number of hydrogen bonds fluctuated within a relatively stable range of approximately 130–190, indicating consistent interactions throughout the simulation, with the average number of hydrogen bonds being around 160 (Figure 12a). Furthermore, the Nirmatrelvir–HDAC3 complex displayed a similar trend, maintaining a dense and steady hydrogen bonding pattern within the 140–200 range, which suggests a strong and stable binding interface with average hydrogen bonds of around 170 (Figure 12b). In contrast, the Nirmatrelvir–STAT3 complex showed a significantly lower number of hydrogen bonds, fluctuating roughly between 15 and 45, implying the presence of weaker or more transient interactions compared to the HDAC complexes (Figure 12c). The consistent patterns of hydrogen bonds were reported in the repeated simulations for HDAC1, HDAC3, and STAT3, confirming the reproducibility of the results. Overall, the data suggest that Nirmatrelvir forms more stable hydrogen bonding networks with HDAC1 and HDAC3 than with STAT3, indicating its stronger and potentially more favorable binding to the HDAC targets.

### 2.10. Binding Free Energy Calculation of Nirmatrelvir–Protein Complex

Post-simulation binding free energy analyses, which consider the dynamic behavior of molecules observed during simulations, play a vital role in accurately estimating the strength of drug–protein interactions. Unlike static docking scores, approaches like MM/GBSA and MM/PBSA break down the binding energy into specific components, such as van der Waals interactions, electrostatic forces, and solvation energies. This breakdown helps reveal the individual forces stabilizing the complex, offering a deeper understanding of molecular binding mechanisms [50]. By elucidating thermodynamic profiles, these calculations help rank lead compounds, validate docking predictions, and direct chemical optimization, guaranteeing that only the most promising compounds progress, ultimately increasing drug discovery efficiency [51]. Consequently, we employed the MM/GBSA and MM/PBSA methods to calculate the binding free energy of our drug–protein complexes. The binding free energy analysis of Nirmatrelvir with three target proteins, HDAC1, HDAC3, and STAT3, using MM/GBSA and MM/PBSA methods provided valuable insights into the strength and stability of their interactions. According to both approaches, Nirmatrelvir exhibits the most favorable total binding free energy with HDAC3, followed by HDAC1, and the weakest interaction with STAT3. Specifically, the MM/GBSA-derived ∆G_total values were −33.3667 ± 0.50 kcal/mol for Nirmatrelvir–HDAC3, −23.69 ± 0.42 kcal/mol for Nirmatrelvir–HDAC1, and −21.17 ± 0.39 kcal/mol for Nirmatrelvir–STAT3. A similar trend was observed with MM/PBSA, with ∆G_total values of −27.77 ± 0.64 kcal/mol, −17.99 ± 0.56 kcal/mol, and −16.99 ± 0.49 kcal/mol, respectively (Table 3). The dominant contribution in all complexes was derived from van der Waals interactions (ΔE_vdw), especially in Nirmatrelvir–HDAC3 (−40.03 ± 0.55 kcal/mol), indicating strong hydrophobic contacts. Electrostatic contributions (ΔE_ele) were relatively minor, while solvation energies (both polar and non-polar components) opposed binding to varying degrees, with HDAC3 again showing the least unfavorable solvation penalty. Overall, these results suggest that Nirmatrelvir binds most strongly to HDAC3, moderately to HDAC1, and least effectively to STAT3, supporting the notion that HDAC3 may be the most promising target for Nirmatrelvir.

## 3. Materials and Methods

### 3.1. Prediction of Drug Targets and Disease-Associated Targets

To identify potential targets of Nirmatrelvir in HCC, three drug target prediction platforms were employed: SwissTargetPrediction (http://www.swisstargetprediction.ch/) (accessed on 10 November 2024) [52], SuperPred (https://prediction.charite.de/) (accessed on 10 November 2024) [53], and Way2Drug (https://www.way2drug.com/clc-pred/) (accessed on 10 November 2024) [54]. The Canonical SMILES of Nirmatrelvir was obtained from the ChemSpider database, and queries were made into each database to obtain the pharmacologically relevant targets. Additionally, DisGeNet (https://www.disgenet.org/search) (accessed on 12 November 2024) was used to retrieve genes associated with HCC by searching for the term “hepatocellular carcinoma.” Subsequently, drug-related targets and HCC-associated genes were overlapped to identify common therapeutic targets. The shared proteins were further analyzed and used to construct a protein–protein interaction (PPI) network, which formed the basis for subsequent experimental steps. A visual representation of the overall methodology is provided in Figure 2.

### 3.2. Protein–Protein Interaction Network Analysis of Common Targets

Building a protein–protein interaction (PPI) network enables potential drug targets to be identified by uncovering essential signaling pathways and key proteins involved in disease mechanisms. To investigate the potential cancer-related targets of Nirmatrelvir, a PPI network was generated using the STRING database (https://string-db.org/) (accessed on 25 November 2024), applying a stringent confidence threshold of 0.900 [55,56]. This high-confidence setting ensures that only the most reliable and experimentally supported protein interactions are considered, offering a robust framework for pinpointing key therapeutic candidates. The constructed network was further analyzed using Cytoscape v3.9.1, where the MCODE plug-in was utilized to identify highly interconnected subnetworks, applying the default parameters. Furthermore, to highlight the most influential nodes within the network, CytoHubba was employed to rank the top 10 hub genes based on degree scores [57,58,59]. The selection criteria for hub genes were based on the topological analysis of the protein–protein interaction (PPI) network. Specifically, we applied centrality measures such as degree to rank genes. The genes that consistently ranked highest were designated as hub genes, as they likely play crucial regulatory roles within the network. Among these, the top three genes, selected for their prominent biological roles, were subjected to more detailed investigation, such as survival analysis, molecular docking, and molecular dynamic simulation.

### 3.3. Enrichment Analysis of Top 10 Hub Genes

For the enrichment analysis of the hub genes, we utilized the DAVID v6.8 online platform (https://david.ncifcrf.gov/) (accessed on 27 November 2024). The gene list was uploaded to the server, and various criteria were configured, including species selection, molecular functions, biological processes, cellular component localization, and functional annotations. Pathway and functional enrichment were assessed using resources from the KEGG and Gene Ontology databases [60,61]. A two-sided hypergeometric distribution was applied for statistical analysis, with the Benjamini–Hochberg correction employed to adjust for multiple testing [62].

### 3.4. Survival and Expression Analysis of Selected Genes in Control and Cancer Patients

Gene expression profiling interactive analysis (GEPIA2; http://gepia2.cancer-pku.cn/) (accessed on 7 December 2024) [39] is a powerful web-based resource that integrates gene expression data from both The Cancer Genome Atlas (TCGA) and the Genotype-Tissue Expression (GTEx) projects. This tool facilitates a variety of analyses, including the identification of genes with altered expression in different types of cancer, and enables the investigation of their expression trends and prognostic significance. In the current study, GEPIA2’s single-gene analysis module was utilized to explore how specific gene candidates influence overall survival in hepatocellular carcinoma. The survival analysis incorporated hazard ratios (HRs), 95% confidence intervals, and log-rank *p*-values, following a previously reported methodology [63]. Furthermore, the expression of a selected gene was analyzed in both control and cancer patients.

### 3.5. Retrieval and Preparation of Target Proteins and Nirmatrelvir Structures

In structure-based drug design (SBDD), target protein crystallographic information is central to the understanding of molecular interactions, optimizing drug efficacy, and minimizing off-target effects [64], enabling the design of compounds that are defined by a high binding affinity and specificity [65]. The X-ray crystallographic structures for the selected hub proteins HDAC1 (PDB ID: 4BKX), HDAC3 (PDB ID: 4A69), and STAT3 (PDB ID: 6QHD) were downloaded from the Protein Data Bank (PDB; https://www.rcsb.org/) (accessed on 27 December 2024) [66]. The protein structures were then prepared and energy-minimized using Chimera software (1.15) [67,68] to ensure structural stability. The Nirmatrelvir drug was downloaded from the ChemSpider database (https://www.chemspider.com/) (accessed on 27 December 2024) and its conformation was optimized using the MMFF94 force field to enable effective docking simulations [69].

### 3.6. Molecular Docking of Nirmatrelvir with Selected Hub Genes

The evaluation of the target proteins’ binding interactions with Nirmatrelvir was performed through the induced-fit docking (IFD) technique using AutoDockFR [70]. To increase the specificity of docking, the grid box was generated based on the co-crystalized ligand. The assigned grid box coordinates for HDAC 1 were X = −38.01, Y = 0.97, Z = −6.46. However, in the case of HDAC3, the dimensions were set to X = 32.48, Y = 60.44, and Z = 28.15. Furthermore, the grid box dimensions for STAT3 were set to X = −59.77, Y = −59.11, and Z = −29.65. A ligand was docked to produce 20 different poses, and the most favorable pose was selected based on its binding energy and interaction profiles.

### 3.7. Docking Visualization and Analysis

Post-docking visualization and analysis were conducted using PyMOL (2.5.4) and Schrödinger Maestro (academic version). Additionally, to validate the docking results and examine the stability of the Nirmatrelvir–target protein complexes, molecular dynamics (MD) simulations were performed, providing further insights into the interaction stability [71].

### 3.8. Molecular Dynamics Simulations of Drug–Protein Complexes

To complement the confirmation of the drug–protein complex’s stability, assessed through molecular docking, molecular dynamics (MD) simulations were performed using the AMBER22 package [72]. The preparation of coordinate and topology files for each molecular system was carried out using the tLeap module integrated within the AMBER 21 software suite. To mimic a realistic aqueous environment, each system was solvated in an Optimal Point Charge (OPC) water box, with counterions added to neutralize the overall charge. Ligand parameters were generated using the Antechamber and Parmchk2 utilities, applying the GAFF2 force field to assign appropriate atom types and force field parameters. Prior to running 200 ns molecular dynamics simulations, all systems underwent energy minimization using a combination of the steepest descent and conjugate gradient algorithms to relieve steric clashes and unfavorable contacts. Controlled heating protocols were applied to gradually increase the system’s temperature to the desired value, using either Langevin dynamics or the Berendsen thermostat to regulate thermal conditions [73]. Non-bonded interactions were treated using the Lennard-Jones potential for van der Waals forces, while long-range electrostatics were handled with the particle mesh Ewald (PME) approach [74]. The systems were equilibrated after minimization and heating using a sequence of steps: restrained minimization, temperature ramping, and unrestrained equilibration to achieve thermal and pressure stability.

### 3.9. Post-Simulation Analysis of Drug–Protein Complexes

To analyze the binding stability of the Nirmatrelvir–protein complexes in a dynamic environment, we calculated interaction-based parameters such as RMSD (Root Mean Square Deviation), Rg (Radius of Gyration), and RMSF (Root Mean Square Deviation) for each complex. By using the NPT (constant pressure) or NVT (constant volume), we performed a 200 ns molecular dynamics simulation [72]. Furthermore, the CPPTRAJ module was used to process the post-simulation trajectories. This evaluation offered a deeper understanding of how the drug interacted with the protein’s active sites over time, highlighting key aspects such as structural flexibility and binding stability. The following equations were used to calculate the parameters.

(1) $$RMSD=\sqrt{\frac{\sum d^{2}i=1}{N_{atoms}}}$$

(2) Thermal factor or B−factor=[(8π∗∗2)/3](msf)

The Radius of Gyration serves as a proxy for the degree of structural compactness in proteins.

(3) $$R_{gyr}^{2}=\frac{1}{M}\;\sum_{i=1}^{N}m_{i}\left(r_{i}-R^{2}\right)$$

where

(4) $$M=\sum_{i=1}^{N}m_{i}$$

(5) $$R=N^{-1}\sum_{i=1}^{N}r_{i}$$

### 3.10. Estimating Binding Free Energy Using MM/GBSA and MM/PBSA Approaches

The MM/GBSA and MM/PBSA approaches were used to calculate the binding free energies between Nirmatrelvir and the selected proteins to measure the binding strength. These methods are well known for being more economical and computationally efficient than resource-intensive ones [75]. For each complex, the binding free energy was estimated by evaluating the solvation energies of the complete complex, the unbound target protein, and the free Nirmatrelvir molecule. These individual energy values were then integrated to calculate the overall binding affinity. Each term in the total binding energy was computed using the following equation.

(6) $${∆G}_{\mathrm{bind}}=G_{\left(\mathrm{complex},\mathrm{solvated}\right)}-G_{\left(\mathrm{Paxlovid},\mathrm{solvated}\right)}-G_{\left(\mathrm{receptors},\mathrm{solvated}\right)}$$

The contribution of the interaction in the complex can be ascertained using Equation (7):

(7) $$G=E_{\mathrm{Molecular}\mathrm{Mechanics}}-G_{\mathrm{solvated}}-TS$$

To determine the precise energy term, this equation can be further rearranged.

(8) $${∆G}_{\mathrm{bind}}={∆E}_{\mathrm{Molecular}\mathrm{Mechanics}}+{∆G}_{\mathrm{solvated}}-∆TS={∆G}_{\mathrm{vaccum}}+{∆G}_{\mathrm{solvated}}$$

(9) $${∆E}_{\mathrm{Molecular}\mathrm{Mechanics}}={∆E}_{\mathrm{int}}+{∆E}_{\mathrm{electrostatic}}+{∆E}_{\mathrm{vdW}}$$

(10) $$∆G_{\mathrm{solvated}}=∆G_{\mathrm{Generalized}\mathrm{born}}+∆G_{\mathrm{surface}\mathrm{area}}$$

(11) $$∆G_{\mathrm{surface}\mathrm{area}}=\gamma .SASA+b$$

(12) $$∆G_{\mathrm{vaccum}}={∆E}_{\mathrm{Molecular}\mathrm{Mechanics}}-T∆S$$

## 4. Conclusions

This study systematically employed integrative network pharmacology, molecular docking, and molecular dynamics simulations to investigate the potential repurposing of Nirmatrelvir as a therapeutic agent for HCC. HDAC1, HDAC3, and STAT3 were identified as key hub proteins implicated in HCC pathogenesis with significant binding interactions with Nirmatrelvir. Among these, HDAC3 demonstrated the most favorable interactions, as indicated by docking scores (−6.026 kcal/mol), dynamic stability, and binding free energy values derived from MM/GBSA (−33.3667 ± 0.50 kcal/mol) and MM/PBSA (−27.7671 ± 0.64 kcal/mol) calculations. Our integrative in silico analysis revealed that Nirmatrelvir, which was originally developed as an antiviral agent, may exhibit previously unrecognized inhibitory activity against HDAC1 and HDAC3, which are critical epigenetic regulators in HCC. These findings highlight a promising avenue for the repurposing of drugs and epigenetic interventions in liver cancer. Moreover, these findings provide a rationale for further investigation into Nirmatrelvir’s anticancer potential, reinforcing the broader therapeutic value of repurposing existing antivirals in oncology. These findings provide a foundation for the further exploration of Nirmatrelvir’s anticancer properties and underscore the value of its repurposing potential in cancer. Despite providing valuable insights, the computational nature of this study does not fully replicate the complexity of biological systems. Therefore, experimental validation through in vitro and in vivo studies is necessary to confirm Nirmatrelvir’s therapeutic efficacy and elucidate its mechanistic role in hepatocellular carcinoma.

## Figures and Tables

**Table 1:** Distribution of top 10 hub genes in biological processes, cellular components, molecular functions, and KEGG pathways based on FDR significance value.

GO ID	Description of Terms	Matching Proteins in the Network	False Discovery Rate (FDR)
Go Biological Processes			
GO:0006476~	protein deacetylation	HDAC3, HDAC1, HDAC6	0.004203419
GO:0040029~	epigenetic regulation of gene expression	HDAC5, HDAC3, HDAC6	0.027060077
GO:0000165~	MAPK cascade	AR, PRKCE, PTK2B	0.088593245
GO:2000273~	positive regulation of signaling receptor activity	HDAC1, HDAC6	0.204044592
GO:0006468~	protein phosphorylation	PRKCE, PRKCD, PTK2B	0.224965856
GO:0080090~	regulation of primary metabolic process	PRKCE, PRKCD	0.224965856
GO:0051279~	regulation of release of sequestered calcium ions into cytosol	PRKCE, PTK2B	0.224965856
GO:0032930~	positive regulation of superoxide anion generation	PRKCE, PRKCD	0.259158844
GO:0040014~	regulation of multicellular organism growth	HDAC3, STAT3	0.261175273
GO:0035902~	response to immobilization stress	PTK2B, HDAC6	0.277327388
GO:0030522~	intracellular receptor signaling pathway	AR, STAT3	0.304683589
GO:0051968~	positive regulation of synaptic transmission, glutamatergic	PTK2B, HDAC6	0.304683589
GO:0010634~	positive regulation of epithelial cell migration	PRKCE, HDAC6	0.318624474
GO:0042307~	positive regulation of protein import into nucleus	HDAC3, PRKCD	0.33956092
GO:0010628~	positive regulation of gene expression	AR, HDAC1, STAT3	0.33956092
GO:0043066~	negative regulation of apoptotic process	HDAC3, HDAC1, PTK2B	0.33956092
GO:0008284~	positive regulation of cell population proliferation	AR, HDAC1, PTK2B	0.33956092
**Go Cellular Components**			
GO:0005829~	cytosol	HDAC5, AR, HDAC3, HDAC1, PRKCE, STAT3, PRKCD, PTK2B, HDAC6	0.005745823
GO:0005737~	cytoplasm	HDAC5, AR, HDAC3, HDAC1, PRKCE, STAT3, PRKCD, PTK2B, HDAC6	0.005810795
GO:0005634~	nucleus	HDAC5, AR, HDAC3, HDAC1, PRKCE, STAT3, PRKCD, PTK2B, HDAC6	0.005999562
GO:0005654~	nucleoplasm	HDAC5, AR, HDAC3, HDAC1, STAT3, PRKCD, HDAC6	0.030761078
GO:0005886~	plasma membrane	AR, HDAC3, PRKCE, STAT3, PRKCD, PTK2B, PLG	0.106703595
GO:0005794~	Golgi apparatus	HDAC5, HDAC3, PRKCE	0.43544037
**Go Molecular Function**			
GO:0019899~	enzyme binding	AR, HDAC3, HDAC1, PRKCE, PRKCD, PLG, HDAC6	1.14 × 10^−7^
GO:0033558~	protein lysine deacetylase activity	HDAC5, HDAC3, HDAC1, HDAC6	1.80 × 10^−7^
GO:0004407~	histone deacetylase activity	HDAC5, HDAC3, HDAC1, HDAC6	5.49 × 10^−7^
GO:0001222~	transcription corepressor binding	HDAC5, HDAC3, HDAC1, HDAC6	1.00 × 10^−5^
GO:0042903~	tubulin deacetylase activity	HDAC3, HDAC1, HDAC6	2.73 × 10^−5^
GO:0042826~	histone deacetylase binding	HDAC5, HDAC3, HDAC1, HDAC6	1.49 × 10^−4^
GO:0140297~	DNA-binding transcription factors	HDAC5, HDAC3, HDAC1, STAT3	3.26 × 10^−4^
GO:0061629~	RNA polymerase II-specific DNA-binding transcription factors	HDAC5, AR, HDAC1, STAT3	3.71 × 10^−4^
GO:0160216~	protein lysine deacetylase activity	HDAC3, HDAC1	0.006927449
GO:0000978~	RNA polymerase II cis-regulatory region sequence-specific DNA binding	HDAC5, AR, HDAC1, STAT3, HDAC6	0.006927449
GO:0160008~	protein decrotonylase activity	HDAC3, HDAC1	0.00788652
GO:0160009~	histone decrotonylase activity	HDAC3, HDAC1	0.00788652
GO:0000976~	transcription cis-regulatory region binding	HDAC5, AR, STAT3	0.021646237
GO:0004699~	diacylglycerol-dependent, calcium-independent serine/threonine kinase activity	PRKCE, PRKCD	0.025483135
**KEGG Pathways**			
hsa05203:	viral carcinogenesis	HDAC5, HDAC3, HDAC1, STAT3, HDAC6	0.002396235
hsa05206:	microRNAs in cancer	HDAC5, HDAC1, PRKCE, STAT3	0.058065466
hsa04933:	AGE-RAGE signaling pathway in diabetic complications	PRKCE, STAT3, PRKCD	0.063717187
hsa04931:	insulin resistance	PRKCE, STAT3, PRKCD	0.063717187
hsa04062:	chemokine signaling pathway	STAT3, PRKCD, PTK2B	0.164458095
hsa04930:	type II diabetes mellitus	PRKCE, PRKCD	0.438158384
hsa04912:	GnRH signaling pathway	PRKCD, PTK2B	0.613147394
hsa04750:	inflammatory mediator regulation of TRP channels	PRKCE, PRKCD	0.613147394
hsa04666:	Fc-gamma R-mediated phagocytosis	PRKCE, PRKCD	0.613147394
hsa05200:	pathways in cancer	AR, HDAC1, STAT3	0.613147394

**Table 2:** Molecular docking of Nirmatrelvir with HDAC1, HDAC3, and STAT3.

Complex	Docking Score	Ligand Functional Group	P300 (1P4Q)	Interaction	Distance (Å)
*HDAC1–Nirmatrelvir complex*	−7.319	NH(pyrrolidin-2-one)	Tyr201	HB	2.06
		CO(pyrrolidin-2-one)	Thr208	HB	2.39
		CO(pyrrolidin-2-one)	Gly209	HB	2.10
		CO(pyrrolidin-2-one)	Asp210	HB	2.17
		NH(amide)	Asp213	HB	2.00
*HDAC3–Nirmatrelvir complex*	−6.026	CO(trifluoroacetamide)	Asp93	HB	1.92
		CO(cyanoethyl)acetamide	Hie172	HB	1.93
		NH(pyrrolidin-2-one)	Gly267	HB	2.05
*STAT3–Nirmatrelvir complex*	−6.304	CO(cyanoethyl)acetamide	Lys591	HB	2.05
		N(cyanoethyl)acetamide	Lys591	HB	2.41
		N(cyanoethyl)acetamide	Arg609	HB	2.24
		N(cyanoethyl)acetamide	Ser630	HB	1.83
		CO(amide)	Ser630	HB	2.31
		NH(pyrrolidin-2-one)	Ser613	HB	1.92
		CO(trifluoroacetamide)	Gln627	HB	1.74

**Table 3:** List of binding free energies of Nirmatrelvir–protein complexes calculated using MM/GBSA and MM/PBSA approaches.

MM/GBSA			
Parameters	Nirmatrelvir–HDAC1	Nirmatrelvir–HDAC3	Nirmatrelvir–STAT3
ΔEvdw	−31.5788 ± 0.61	−40.0338 ± 0.55	−27.4311 ± 0.38
ΔEele	−2.4940 ± 0.40	−0.9344 ± 0.28	−2.2372 ± 0.37
EGB	14.2238 ± 0.52	12.4600 ± 0.36	11.4875 ± 0.33
ESURF	−3.8437 ± 0.08	−4.8585 ± −0.05	−2.9870 ± 0.03
Delta G Gas	−34.0728 ± 0.71	−40.9682 ± 0.75	−29.6683 ± 0.63
Delta G Solv	10.3801 ± 0.47	7.6014 ± 0.32	8.5005 ± 0.32
**∆G total**	**−23.6927 ± 0.42**	**−33.3667 ± 0.50**	**−21.1677 ± 0.39**
MM/PBSA			
Parameters	Nirmatrelvir–HDAC1	Nirmatrelvir–HDAC3	Nirmatrelvir–STAT3
ΔEvdw	−31.5788 ± 0.61	−40.0338 ± 0.55	−27.4311 ± 0.38
ΔEele	−2.4940 ± 0.40	−0.9344 ± 0.28	−2.2372 ± 0.37
EPB	19.0255 ± 0.84	16.6451 ± 0.58	15.0775 ± 0.45
ENPOLAR	−2.9402 ± 0.05	−3.4440 ± 0.02	−2.3957 ± 0.02
Delta G Gas	−34.0728 ± 0.71	−40.9682 ± 0.75	−29.6683 ± 0.63
Delta G Solv	16.0853 ± 0.80	13.2011 ± 0.56	12.6818 ± 0.44
**∆G total**	**−17.9875 ± 0.56**	**−27.7671 ± 0.64**	**−16.9865 ± 0.49**

## Data Availability

The original contributions presented in this study are included in the article. Further inquiries can be directed to the corresponding authors.
